# AIDA (Artificial Intelligence Dystocia Algorithm) in Prolonged Dystocic Labor: Focus on Asynclitism Degree

**DOI:** 10.3390/jimaging10080194

**Published:** 2024-08-09

**Authors:** Antonio Malvasi, Lorenzo E. Malgieri, Ettore Cicinelli, Antonella Vimercati, Reuven Achiron, Radmila Sparić, Antonio D’Amato, Giorgio Maria Baldini, Miriam Dellino, Giuseppe Trojano, Renata Beck, Tommaso Difonzo, Andrea Tinelli

**Affiliations:** 1 Department of Interdisciplinary Medicine (DIM), Unit of Obstetrics and Gynecology, University of Bari “Aldo Moro”, Policlinico of Bari, Piazza Giulio Cesare 11, 70124 Bari, Italy; antoniomalvasi@gmail.com (A.M.); ettore.cicinelli@uniba.it (E.C.); antoniodamato19@libero.it (A.D.); gbaldini97@gmail.com (G.M.B.); miriam.dellino@uniba.it (M.D.); difonzo.tommaso.md@gmail.com (T.D.); 2 Chief Innovation Officer in CLE, 70126 Bari, Italy; lorenzo@malgieri.org; 3 Department of Precision and Regenerative Medicine and Jonic Area, University of Bari “Aldo Moro”, 70121 Bari, Italy; antonella.vimercati@uniba.it; 4 Sackler Faculty of Medicine, Tel-Aviv University, Tel-Aviv 6997801, Israel; reuvenachiron@gmail.com; 5 Clinic for Gynecology and Obstetrics, University Clinical Centre of Serbia, 11000 Belgrade, Serbia; 6 Faculty of Medicine, University of Belgrade, 11000 Belgrade, Serbia; 7 Department of Maternal and Child Gynecologic Oncology Unit, “Madonna delle Grazie” Hospital ASM, 75100 Matera, Italy; giutrojano@gmail.com; 8 Department of Medical and Surgical Sciences, Anesthesia and Intensive Care Unit, Policlinico Riuniti Foggia, University of Foggia, 71122 Foggia, Italy; beckrenata64@gmail.com; 9 Department of Obstetrics and Gynecology and CERICSAL (CEntro di RIcerca Clinico SALentino), Veris delli Ponti Hospital Scorrano, 73020 Lecce, Italy

**Keywords:** artificial intelligence, intrapartum ultrasound, dystocia, asynclitism, labor, cesarean section, vaginal operative delivery, malrotation, malposition

## Abstract

Asynclitism, a misalignment of the fetal head with respect to the plane of passage through the birth canal, represents a significant obstetric challenge. High degrees of asynclitism are associated with labor dystocia, difficult operative delivery, and cesarean delivery. Despite its clinical relevance, the diagnosis of asynclitism and its influence on the outcome of labor remain matters of debate. This study analyzes the role of the degree of asynclitism (AD) in assessing labor progress and predicting labor outcome, focusing on its ability to predict intrapartum cesarean delivery (ICD) versus non-cesarean delivery. The study also aims to assess the performance of the AIDA (Artificial Intelligence Dystocia Algorithm) algorithm in integrating AD with other ultrasound parameters for predicting labor outcome. This retrospective study involved 135 full-term nulliparous patients with singleton fetuses in cephalic presentation undergoing neuraxial analgesia. Data were collected at three Italian hospitals between January 2014 and December 2020. In addition to routine digital vaginal examination, all patients underwent intrapartum ultrasound (IU) during protracted second stage of labor (greater than three hours). Four geometric parameters were measured using standard 3.5 MHz transabdominal ultrasound probes: head-to-symphysis distance (HSD), degree of asynclitism (AD), angle of progression (AoP), and midline angle (MLA). The AIDA algorithm, a machine learning-based decision support system, was used to classify patients into five classes (from 0 to 4) based on the values of the four geometric parameters and to predict labor outcome (ICD or non-ICD). Six machine learning algorithms were used: MLP (multi-layer perceptron), RF (random forest), SVM (support vector machine), XGBoost, LR (logistic regression), and DT (decision tree). Pearson’s correlation was used to investigate the relationship between AD and the other parameters. A degree of asynclitism greater than 70 mm was found to be significantly associated with an increased rate of cesarean deliveries. Pearson’s correlation analysis showed a weak to very weak correlation between AD and AoP (PC = 0.36, *p* < 0.001), AD and HSD (PC = 0.18, *p* < 0.05), and AD and MLA (PC = 0.14). The AIDA algorithm demonstrated high accuracy in predicting labor outcome, particularly for AIDA classes 0 and 4, with 100% agreement with physician-practiced labor outcome in two cases (RF and SVM algorithms) and slightly lower agreement with MLP. For AIDA class 3, the RF algorithm performed best, with an accuracy of 92%. AD, in combination with HSD, MLA, and AoP, plays a significant role in predicting labor dystocia and labor outcome. The AIDA algorithm, based on these four geometric parameters, has proven to be a promising decision support tool for predicting labor outcome and may help reduce the need for unnecessary cesarean deliveries, while improving maternal-fetal outcomes. Future studies with larger cohorts are needed to further validate these findings and refine the cut-off thresholds for AD and other parameters in the AIDA algorithm.

## 1. Introduction

Asynclitism is a critical concept in obstetrics, referring to an abnormal position of the fetal head as it enters and progresses through the birth canal. In a normal labor, the fetal head descends through the maternal pelvis with its sagittal suture aligned with the anteroposterior diameter of the pelvis. The term “asynclitism” specifically describes a situation where the fetal head is tilted laterally, causing one parietal bone to present before the other. Where the anterior parietal bone presents first, the condition is identified as anterior asynclitism; where the posterior parietal bone leads, the condition is described as posterior asynclitism [1]. Around 15% of people experience asynclitism during labor, with anterior asynclitism being more common than posterior asynclitism, according to Hung et al. [2].

The clinical significance of asynclitism lies in its potential to complicate labor progression. Physiological asynclitism refers to the fetal head’s minor “tilted” posture in the birth canal and is a result of the fetal head’s physiological adjustment to the mother’s pelvis throughout labor [3,4]. Mild asynclitism is generally considered a normal adaptation mechanism that helps the fetal head to negotiate the changing dimensions and angles of the maternal pelvis during labor. Malposition and malrotation of the fetal head in the birth canal, characterized by significant or pathological asynclitism, can potentially result in prolonged labor, an increased need for operative interventions, and in some cases, cesarean delivery [5].

Understanding and accurately diagnosing asynclitism is crucial for optimal management of labor, as it can inform decisions about the timing and type of interventions needed. The spectrum of physiological from pathological asynclitism is well recognized; thus, the prevalence of asynclitism still has to be ascertained [6]. 

Digital vaginal examination presents challenges in diagnosing pronounced asynclitism, particularly when it coexists with other malpositions [7]. Caput succedaneum during dystocic labor prevents sutures and fontanelles from being recognized during digital vaginal examination, making the clinical diagnosis of asynclitism more difficult to make, and thus the prevalence of asynclitism is not easily detected [8]. 

Over the past three decades, the integration of ultrasound technology in delivery rooms has significantly enhanced the diagnosis of both asynclitism and other fetal malpositions [9,10]. This advancement has provided clinicians with a more accurate and objective method for assessing fetal head position and alignment during labor, overcoming many of the limitations associated with traditional digital examination techniques. Intrapartum ultrasound has particularly improved the detection and quantification of asynclitism, allowing for more precise evaluation of its degree and potential impact on labor progress. To minimize complications for both mother and fetus [11], accurate diagnosis and precise measurement of the degree of asynclitism are crucial for guiding appropriate management decisions, particularly in cases of pronounced asynclitism, and especially when considering operative vaginal delivery or cesarean section [12]. If asynclitism is diagnosed during prolonged dystocic labor under neuraxial analgesia, it is more crucial to decide on the delivery mode and stop using analgesic medications. 

The measurement of asynclitism degree plays a crucial role in defining “geometric dystocia” through the Artificial Intelligence Dystocia Algorithm (AIDA) [13]. This innovative approach combines the ultrasound measures of four geometric parameters: asynclitism degree (AD), angle of progression (AoP), midline angle (MLA), and head-symphysis distance (HSD) to provide a more comprehensive assessment of labor progress and delivery outcome. The AD measurement quantifies the extent to which the fetal head is tilted relative to the birth canal. This information is vital because significant asynclitism can impede labor progress and increase the likelihood of operative vaginal delivery (operative VD) or intrapartum cesarean delivery (ICD).

From a geometric perspective, the four parameters work in complementary ways to provide a comprehensive three-dimensional assessment of the fetus’s position and progression during labor. The AD, the AoP, and the MLA collectively define the fetal head’s orientation and alignment within the maternal pelvis. The HSD quantifies the fetal head distance. Together, these parameters create a detailed three-dimensional representation of the fetus’s status and progress, offering a more complete and accurate picture than any single measurement alone. 

This paper aims to highlight the role of asynclitism degree measurement in the development and implementation of AIDA. AIDA represents an innovative approach to labor management that incorporates a human-in-the-loop methodology. This approach utilizes artificial intelligence (AI) algorithms designed to support and enhance physician decision-making, rather than replace it. By prioritizing the clinician’s expertise and judgment, AIDA integrates the principles of explainable artificial intelligence (XAI), ensuring that the AI’s recommendations are transparent, interpretable, and aligned with clinical reasoning. The study emphasizes how the quantification of asynclitism degree contributes to AIDA’s ability to provide valuable insights while maintaining the essential role of human clinical expertise in labor and delivery management.

## 2. Materials and Methods

This retrospective study utilized data from the obstetrics and gynecology departments of three Italian hospitals: “Santa Maria” Hospital, “Vito Fazzi” Hospital, and “Veris Delli Ponti” Hospital, covering the period from January 2014 to December 2020. The Institutional Review Board approved the study (CER 0320), which adhered to the Declaration of Helsinki principles. Patient anonymity was maintained through the use of de-identified data.

We focused on full-term nulliparous women with singleton, cephalic-presenting fetuses who received neuraxial analgesia and intrapartum ultrasound (IU) during a protracted second stage of labor. According to the American College of Obstetricians and Gynecologists (ACOG), a protracted second stage for nulliparous patients with neuraxial analgesia is defined as lasting more than three hours [14,15].

Exclusion criteria encompassed breech, transverse, or oblique presentations; twin pregnancies; abnormal placental implantation; HELLP syndrome; coagulation disorders; uterine hyperstimulation (often referred to as “terrified uterus”); non-reassuring fetal heart rate; thick meconium; and cephalopelvic disproportion.

Patients consented to the anonymous use of their data for research purposes by completing a specific form in their medical file. Obstetricians and midwives performed and recorded digital vaginal examinations during labor and delivery. We collected and anonymized the following data: age, gestational age, body mass index, neonatal weight, Apgar scores at 1 and 5 min, cephalic presentation type (occiput anterior, posterior, or transverse), asynclitism type (anterior or posterior), maternal complications, and delivery outcome (intrapartum cesarean, operative vaginal, or spontaneous delivery).

All patients underwent intrapartum ultrasound (IU) at the three-hour mark of the second stage of labor. Using standard 3.5 MHz transabdominal ultrasound probes, was measured four geometric parameters for each patient: (1) fetal head-symphysis distance (HSD), (2) asynclitism degree (AD), (3) angle of progression (AoP), and (4) midline angle (MLA). During this assessment, we also determined whether the asynclitism was anterior or posterior.

Malvasi and Malgieri et al., in their study referred to as AIDA 1 [13], addressed the challenge of predicting delivery outcomes, ICD or non-ICD, based on four geometric parameters. 

**Figure 1:**
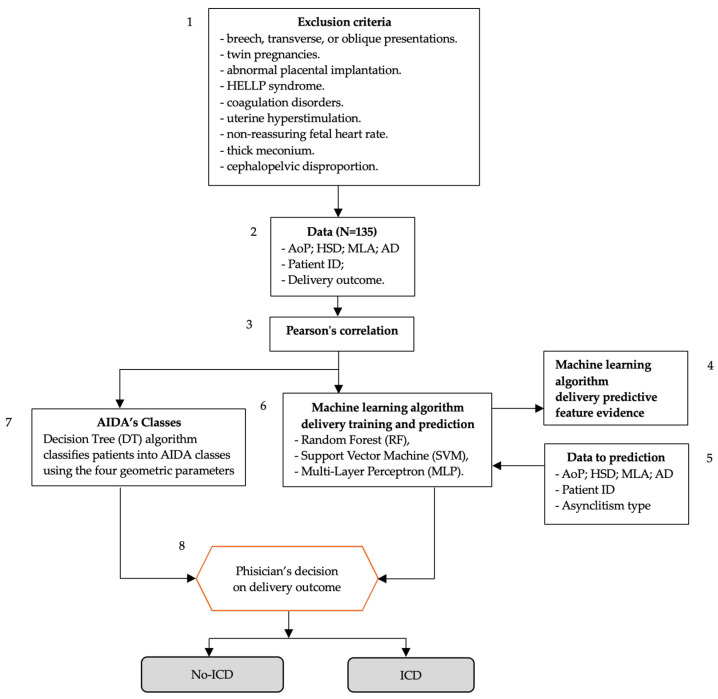
Flowchart of AIDA (Artificial Intelligence Dystocia Algorithm) using the common structure of evidence-based, clinical intrapartum care algorithms defined by the WHO Intrapartum Care Algorithm Working Group [16].

The workflow of the AIDA on predicting delivery outcomes using machine learning algorithms and geometric parameters measured during labor is illustrated in Figure 1. The flowchart utilizes boxes that align with those defined by the WHO Intrapartum Care Algorithm Working Group [16]. The process is as follows: (1) exclusion criteria are listed at the top, detailing conditions that would exclude patients from the study; (2) data collection (N = 135) includes four geometric parameters (AoP, HSD, MLA, AD), patient ID, and delivery outcome; (3) Pearson’s correlation is performed on the collected data. The process then splits into two parallel tracks: the first being AIDA’s Classes (5), where a decision Tree (DT) algorithm classifies patients into AIDA classes using the four geometric parameters; and the second being machine learning algorithm delivery training and prediction (6), which uses random forest (RF), support vector machine (SVM), and multi-layer perceptron (MLP) algorithms defined in AIDA [13]. Both tracks feed into (8), the “physician’s decision on delivery outcome” step. The final outcomes are categorized as either no-ICD (no intrapartum cesarean delivery) or ICD (intrapartum cesarean delivery). There are additional elements showing (4) machine learning algorithm delivery predictive feature evidence; and (5) data used for prediction (AoP, HSD, MLA, AD, patient ID, and asynclitism type).

This flowchart demonstrates how AIDA combines clinical data, statistical analysis, and machine learning to predict delivery outcomes and support clinical decision-making.

This paper introduces an advanced iteration of the Artificial Intelligence Dystocia Algorithm, building on the successful outcomes of AIDA [13], specifically designed to delve deeper into the significance of AD and its types (anterior or posterior) in predicting labor outcomes. The iteration involves the following 4 steps:

(a) Correlation analysis: Pearson’s correlation was used to measure the linear relationship between AD and other parameters, verifying their statistical correlation and significance.
(b) Feature importance assessment: the importance of AD as a feature in the three supervised machine learning algorithms applied to the four geometric parameters and physician-determined delivery outcomes was evaluated.
(c) AIDA class definition: The role of AD cutoff values, in conjunction with the other three geometric parameters, in defining AIDA classes was identified. These cutoffs were associated with either intrapartum cesarean delivery (ICD) or non-ICD outcomes.
(d) Delivery outcome and asynclitism type analysis: The role of asynclitism type (anterior or posterior) in delivery outcomes was examined, and potential scenarios physicians might encounter when using AIDA to predict delivery outcomes were highlighted.

This structured approach aimed to provide a comprehensive understanding of asynclitism’s impact on labor progression and delivery outcomes.

### 2.1. AD and Pearson’s Correlation

In the first step, the relationship between AD and other key parameters was investigated using Pearson’s correlation analysis. The parameters examined included the AoP, the HSD, and the MLA. Additionally, we analyzed correlations between AD and Apgar scores at 1 and 5 min. Pearson’s coefficients quantify correlation strength and direction, ranging from −1 to +1. Values indicate: ±0.00–0.19 (very weak), ±0.20–0.39 (weak), ±0.40–0.59 (moderate), ±0.60–0.79 (strong), or ±0.80–1.00 (very strong). *p*-values determine statistical significance (*p* < 0.05 *, *p* < 0.01 **, *p* < 0.001 ***), avoiding ambiguous terms. This method provides clear, standardized correlation interpretation.

### 2.2. AD as a Predictive Feature in AIDA Machine Learning Algorithms

The second step focused on quantifying the feature importance of asynclitism degree (AD) within the AIDA machine learning algorithms. These algorithms were designed to classify and predict delivery outcomes, distinguishing between intrapartum cesarean delivery (ICD) and non-cesarean Delivery (including operative vaginal and spontaneous deliveries). While all four geometric parameters (AD, angle of progression, head-symphysis distance, and midline angle) served as inputs, we specifically analyzed AD’s relative importance in the predictive models. By calculating AD’s feature importance scores across different algorithms, we aimed to precisely determine its unique contribution to the accuracy and reliability of AIDA’s delivery outcome predictions. This analysis provided crucial insights into asynclitism’s role in labor progression and its predictive power for delivery outcomes within the AIDA framework.

### 2.3. AD and AIDA Classes 

The third step was focused on the role of AD in identifying the thresholds of the four geometric parameters associated with different delivery outcomes. A decision tree (DT) algorithm was chosen for its interpretability and classification suitability, and each parameter pair was analyzed: AoP–AD, HSD–AD, and MLA–AD. For AD, specific cut-off values or ranges distinguishing between ICD and non-ICD outcomes were determined. The focus was on the three AIDA [13] classes: (a) AIDA class 0, with all parameters, including AD, in the green zone; (b) AIDA class 3, with three parameters in the red/yellow zone, and one in the green zone; and (c) AIDA class 4, with all parameters, including AD, in the red/yellow zones.

### 2.4. AD and AIDA Machine Learning Algorithm Delivery Prediction

In the fourth step, we employed three machine learning algorithms: random forest (RF), support vector machine (SVM), and multi-layer perceptron (MLP), previously identified as the best performers in AIDA [13] for predicting delivery outcomes. We generated five different random samples using a 70–30 split: 70% of data (95 patients) for training, and 30% (40 patients) for testing and validation.

Each patient’s record included the delivery outcome, AIDA class identifier, and asynclitism type (anterior or posterior). We evaluated the performance of each algorithm across the three AIDA classes (0, 3, and 4) and recorded predictions for every patient in the random samples. This approach allowed for a comprehensive assessment of the algorithms’ efficacy in various scenarios, considering both AIDA class and asynclitism type.

This structured evaluation provides insights into how well each algorithm performs in predicting delivery outcomes across different AIDA classes and asynclitism types, enhancing our understanding of the AIDA method’s predictive capabilities.

AI analysis was conducted using AlterixDesigner with AlterixAI (V 2023.2.1.89) and IBM SPSS Statistics (V 29.0.2.0). AlterixAI’s ‘create samples tool’ generated five 70–30 split samples. The random seed parameter (1–1000) was adjusted to vary data distribution between estimation (70%) and validation (30%) samples. A default value of 1 and additional values of 0, 250, 500, and 750 were used. IBM SPSS implemented the multi-layer perceptron (MLP) neural network, while AlterixDesigner executed other algorithms. 

## 3. Results

The study included 135 patients with the following demographics: mean age 31.62 years (SD 5.28), mean gestational age 40.16 weeks (SD 1.02) or 283.09 days (SD 7.15), mean BMI 27.52 (SD 2.95), mean neonatal weight 3926.68 g (SD 309.66), and mean Apgar scores at 1 and 5 min of 6.65 (SD 1.22) and 8.74 (SD 1.12), respectively (Table 1). The four geometric parameters measured were: head-symphysis distance (HSD), ranging from 10 to 51 mm (mean 21.47, SD 9.265); asynclitism degree (AD), ranging from 4 to 95 mm (mean 60.18, SD 18.866); midline angle (MLA), ranging from 26° to 90° (mean 62.59, SD 14.986); and angle of progression (AoP), ranging from 72° to 192° (mean 122.75, SD 27.454). The asynclitism type was anterior in 50 patients, and posterior in 85 patients (Table 2).

### 3.1. Findings from AD and Pearson’s Correlation

Pearson’s correlation analysis revealed a very strong, statistically significant relationship between Apgar scores at 1 and 5 min (PC = 0.8, *p* < 0.001), which was expected given their inherent link. Asynclitism degree (AD) showed varying correlations with other geometric parameters: a weak but significant correlation with angle of progression (PC = 0.36, *p* < 0.001), a very weak but significant correlation with head-symphysis distance (PC = 0.18, *p* < 0.05), and a very weak, non-significant correlation with midline angle (PC = 0.14). AD also demonstrated weak but significant correlations with Apgar scores at 1 min (PC = −0.2, *p* < 0.05) and 5 min (PC = −0.19, *p* < 0.05). These results suggest that while AD has some relationship with other parameters and outcomes, these correlations are generally weak, indicating that AD provides unique information not captured by the other measures. A comprehensive summary of these correlation analyses is presented in Table 3.

The delivery outcomes were distributed as follows: 56 cases of intrapartum cesarean delivery (ICD), 22 cases of intrapartum cesarean delivery after failure (ICD after failure), 31 cases of operative vaginal delivery (OVD), and 26 spontaneous deliveries. 

**Figure 2:**
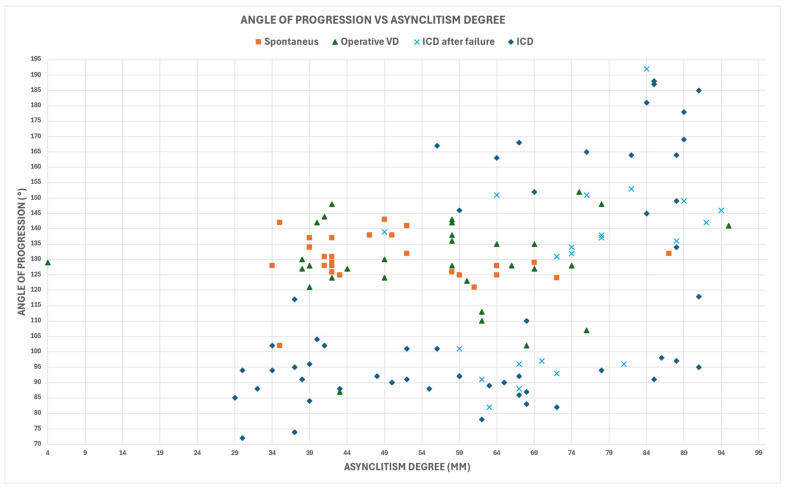
Relationship between asynclitism degree (AD) and angle of progression (AoP) in study cohort (N = 135).

Figure 2 illustrates these outcomes in relation to AD and AoP. The analysis revealed a weak but statistically significant Pearson’s correlation between AD and AoP (*PC* = 0.36, *p*-value < 0.001), emphasizing the complex relationship between these parameters in the context of delivery outcomes.

### 3.2. Findings from AD as a Predictive Feature in AIDA Machine Learning Algorithms

The AIDA study [13] employed six machine learning algorithms for analysis, each revealing a distinct hierarchy of importance for the four geometric parameters. The feature importance rankings of the four geometric parameters varied across algorithms. Multi-layer perceptron (MLP) ranked them as AoP, HSD, MLA, AD. Random Forest (RF) prioritized MLA, HSD, AD, AoP. Support vector machine (SVM) ordered them as HSD, AD, MLA, AoP. XGBoost ranked HSD, AD, and MLA, with AoP not shown. Logistic regression (LR) considered the order of importance to be HSD, MLA, AoP, AD. Decision tree (DT) prioritized MLA, AoP, AD, HSD. These rankings highlight the varying importance of each parameter across different machine learning algorithms.

These varied rankings highlight the complex interplay between the geometric parameters and underscore the importance of using multiple algorithms to capture different aspects of the data. Notably, the position of AD in these rankings provides insight into its relative importance across different modeling approaches. None of the six algorithms considered AD as the top feature in terms of importance when comparing the four geometric parameters (Table 4). Other performance parameters and the ROC curve for each of the six machine learning algorithms used in AIDA are described in [13].

### 3.3. Findings from AD and AIDA Classes

The decision tree (DT) analysis, pairing asynclitism degree (AD) with the other three geometric parameters, yielded distinct cut-off values for predicting intrapartum cesarean delivery (ICD) (Table 5). For AD and angle of progression (AoP), ICD was indicated when AD ≥ 67 mm and AoP ≥ 144.5° or < 101.5°. For AD and head-symphysis distance (HSD), ICD was predicted when AD ≥ 70.5 mm and HSD ≥ 19.5 mm. For AD and midline angle (MLA), ICD was indicated when AD ≥ 65.5 mm.

These results inform the AIDA classes [13] categorization, where ICD is predicted for AD values between 70.5 mm and 95 mm (the maximum in our dataset), while non-ICD is predicted for AD values between 4 mm (the minimum) and 65.5 mm. In our analysis, AD values ≥ 70.5 mm were coded as red (high risk), those between 4 mm and 65.5 mm as green (low risk), and those in the 65.5 mm to 70.5 mm range were considered a cut-off zone and coded as yellow (intermediate risk) [13].

**Figure 3:**
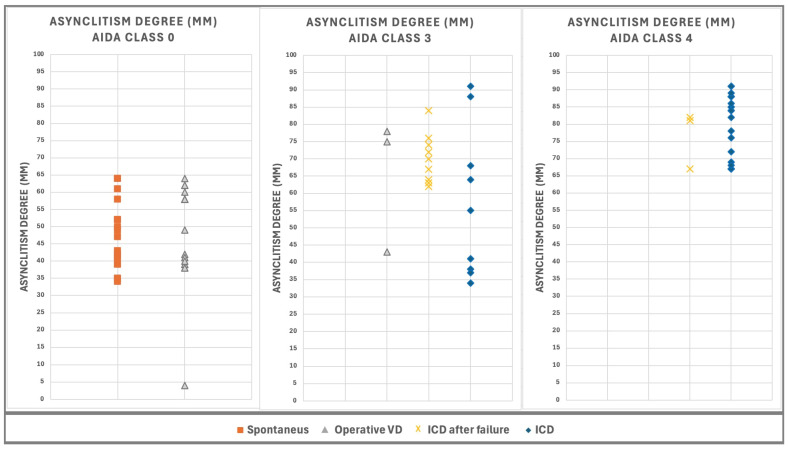
Values of AD, measured at the same time as AoP, HSD, and MLA for 101 of the 135 patient cases involved in the AIDA study [13]. The cases are categorized into three AIDA classes and grouped by delivery outcome. AIDA class 4 (23 cases), AD ranges from 67 mm to 91 mm. AIDA class 3 (38 cases), AD ranges from and 29 mm to 89 mm. AIDA class 0 (40 cases), AD ranges from 4 mm to 64 mm.

The distribution of AD values, measured concurrently with AoP, HSD, and MLA, for 101 out of 135 cases from the AIDA study [13], is illustrated in Figure 3. The cases are categorized into three AIDA classes and further subdivided by asynclitism type. In AIDA class 0, comprising 40 cases with AD ranging from 4 mm to 64 mm, there were 23 cases of anterior asynclitism and 17 cases of posterior asynclitism. AIDA class 3 included 38 cases with AD ranging from 29 mm to 89 mm, consisting of 8 cases of anterior asynclitism and 30 cases of posterior asynclitism. AIDA class 4 contained 23 cases with AD ranging from 67 mm to 91 mm, including 8 cases of anterior asynclitism and 15 cases of posterior asynclitism.

This distribution demonstrates the relationship between AD values, AIDA class categorization, and asynclitism type, providing insight into how these factors correlate with different risk levels for delivery outcomes.

### 3.4. Findings from AD and AIDA Machine Learning Algorithm Delivery Prediction 

As reported in [13], the AIDA method employed a 70–30 split to generate five distinct random samples. Random seed values (1, 0, 250, 500, and 750) were used to vary data row placement. Each resulting 95-record sample was used to predict outcomes for a corresponding 40-record test set. Three top-performing algorithms were applied: random forest (RF), support vector machine (SVM), and multi-layer perceptron (MLP). The five different 70–30 separation samples resulted in 5 groups of 40 predictions each, totaling 200 predictions. Of these, 111 were from distinct patients, while 89 were repetitions due to some patients being selected multiple times by the seeds tool. All predictions were classified according to the AIDA classes.

In the previous study [13], the simultaneous measurement of four geometric parameters enabled the development of AIDA (Artificial Intelligence Dystocia Algorithm). This decision support system offers binary predictions for intrapartum cesarean delivery (ICD) or non-ICD outcomes. AIDA demonstrated high-probability predictions for cases categorized as Class 0 and Class 4, while also providing valuable comparative insights for cases in Class 3. The performance metrics and confusion matrices for the three AIDA machine learning algorithms, where a positive outcome indicates intrapartum cesarean delivery (ICD) and a negative outcome indicates non-ICD, are shown in Table 6.

The results were categorized by AIDA class:

(a) For AIDA class 0, comprising 38 patients with 68 predictions and all four parameters in the green zone, both the random forest (RF) and multi-layer perceptron (MLP) algorithms performed exceptionally well, achieving perfect scores in accuracy, NPV, and specificity, all at 1.0;
(b) In AIDA class 4, which included 18 patients with 31 predictions and all four parameters in the red or yellow zones, the RF and support vector machine (SVM) algorithms demonstrated optimal performance. Both achieved perfect scores in accuracy, PPV, recall, and F1 score, all at 1.0;
(c) For AIDA class 3, consisting of 30 patients with 50 predictions, three parameters in the red or yellow zones, and one parameter in the green zone, the RF algorithm emerged as the top performer. It achieved high accuracy at 0.92, PPV at 0.9167, perfect NPV and recall at 1.0, specificity at 0.3333, and a strong F1 score of 0.9565.

The detailed predictions for each patient across the three AIDA classes are presented in Table 7, Table 8 and Table 9. Table 7 shows results for patients in AIDA class 3, Table 8 for AIDA class 0, and Table 9 for AIDA class 4. In each table, patients are grouped according to their asynclitism type (anterior or posterior).

These tables reflect the outcomes of our prediction model, which utilized five different 70–30 split samples, each composed of 5 groups of 40 predictions. For each patient, we reported every prediction made and the number of times that the patient appeared in the prediction sets. In total, this analysis covered 86 unique patients with 149 predictions across all three AIDA classes.

This comprehensive presentation allows for a detailed examination of the model’s performance across different patient characteristics and AIDA classifications, providing insights into the reliability and consistency of our predictive approach.

## 4. Discussion

### 4.1. Asynclitism, Asynclitism Degree and Other Diagnosis

The four recorded parameters that we analyzed—the MLA, the AoP, the HSD, and the AD—are the most often reported in the literature [17,18]. An ultrasound can be used to assess the fetal position and rotation.

Although a lot of research has been performed on other fetal head malpositions, not much has been focused on asynclitism. Bofil et al. [19] included patients with caput succedaneum and asynclitism degree in a prospective randomized investigation on surgical vaginal delivery with Mcup vacuum extractor vs. forceps and concluded that the vacuum was linked with significantly more cephalohematomas. In a more recent autopsy, Vlasyuk et al. [20] showed how crucial it is to use intrapartum ultrasonography to avoid accidentally using vacuum when there is fetal intracranial injury. When compared to a digital vaginal examination by itself, intrapartum ultrasonography significantly enhanced the diagnosis of asynclitism [21]. The “squint sign”, which is a straightforward and uncomplicated sign to detect using transabdominal ultrasonography, was the first ultrasonography sign reported in the literature by Malvasi et al. [22]. A single orbit is visible when the fetal head is tilted in the birth canal, providing an objective indication that can be photographed and noted during cesarean delivery after lower uterine segment incision [23,24,25].

The translabial technique has been used in the delivery room to diagnose asynclitism, and Malvasi et al. have documented and described additional symptoms in the literature [26]. The discovery of the midline sign was reported by Gimovsky et al. in their study on translabial ultrasonography for the diagnosis of asynclitism [27]. The “anterior midline sign” and the “subpubic midline sign” were identified by Malvasi et al. [28]; posterior asynclitism is diagnosed when the midline shifts toward the pubis. Anterior asynclitism has been diagnosed as the “posterior midline sign”, also known as the “sacral midline sign”, which occurs when the midline is pushed toward the sacrum. Lateral asynclitism [29], a subtype of asynclitism, is identified by a four-chambered image, monstrous profile (caused by prenatal head twisting), and persistent posterior position of the occiput (posterior spine) [30].

Cesarean delivery is necessary as a result of this malposition and malrotation, which is commonly linked to the occiput’s persistent posterior position [31]. Both mechanical [32] and dysfunctional dystocia [33] are caused by marked asynclitism in prolonged dystocic labor. According to American College of Obstetrics and Gynecologists (ACOG) standards, neurovascular changes in the lower uterine segment are caused by fetal head malposition in prolonged labor after three hours, as demonstrated by Malvasi et al. [34,35,36]. The degree of asynclitism is the most crucial factor to consider, particularly in vaginal surgical delivery. Hinkson et al. [37] have reported the significance of intrapartum ultrasonography in rotational forceps use.

Malvasi and Tinelli [38] identified two indicators for diagnosing asynclitism degree in the occiput posterior position: the “squint sign without nose” for high asynclitism and the “squint sign with nose” for medium asynclitism. Using this classification, Birol et al. [39] found that asynclitism without a nose is linked to more surgical deliveries and maternal complications. On the other hand, asynclitism without a nose found by ultrasonography might be helpful in warning obstetricians and averting problems. While this classification allows asynclitism diagnosis, it remains an arbitrary and operator-dependent diagnosis. 

The translabial approach has been used to diagnose asynclitism degree, with scans taken in the sagittal plane or perpendicular to the labia majora. In normal conditions, the parietal diameter and midline are equally spaced, while in asynclitism, cerebral structures are asymmetrical, with the midline shifting either posteriorly (posterior asynclitism) or anteriorly (anterior asynclitism).

Asynclitism developing during head molding changes the cerebral tentorium’s free tension distribution and may cause it to burst. Asynclitism larger than 15 mm increases the risk of brain injury or cerebral tentorium rupture. Both moderate and severe asynclitism are considered pathological due to potential consequences [40].

**Figure 4:**
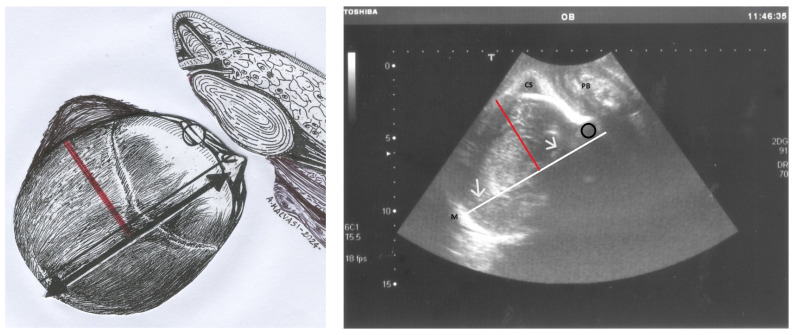
On the left, the drawing shows a longitudinal translabial ultrasound with the fetal head in the right occiput position and anterior asynclitism. The red line represents the degree of anterior asynclitism, measured as the distance between the sagittal suture (depicted by a thick black line) and the left parietal bone. This red line, extending from the midline to the parietal bone, is the ultrasonographic measure of asynclitism degree. On the right, the photo shows the corresponding ultrasound image. The white line is drawn near the midline (M), the hyperechogenic line between the two hemispheres (white arrows). CS: caput succedaneum, located in the left parietal bone; the black circle corresponds to the left anterior squint sign; PS: pubic symphysis.

To make the diagnosis of asynclitism even clearer and more understandable, Figure 4 presents a comparative illustration of anterior asynclitism in fetal head positioning. The left panel features a schematic drawing of a longitudinal translabial ultrasound view. It depicts the fetal head in the right occiput position with anterior asynclitism. A red line demarcates the degree of anterior asynclitism, measured as the distance between the sagittal suture (represented by a thick black line) and the left parietal bone.

The right panel displays the corresponding ultrasound image. Key features include a white line drawn near the midline (M), which appears as a hyperechogenic line separating the two cerebral hemispheres (indicated by white arrows). The caput succedaneum (CS) is visible in the left parietal bone region. A black circle identifies the left anterior squint sign, and the pubic symphysis (PS) serves as an anatomical reference point.

This figure provides a clear visual comparison between the schematic representation and the actual ultrasound image, highlighting the key anatomical landmarks used in identifying and measuring anterior asynclitism.

The International Society of Ultrasound in Obstetrics and Gynecology (ISUOG) guidelines [41] provide level 1 evidence recommending intrapartum ultrasonography before vacuum administration. In cases of malpositions associated with asynclitism, intrapartum ultrasonography has been shown to reduce complications for both mothers [42] and fetuses [43], while also improving the parturients biofeedback [44].

Habek et al. [45] reported that manual rotation techniques were more effective when ultrasonography was used to address asynclitism associated with occiput posterior position. Despite these findings, the relationship between asynclitism degree and other parameters such as head-symphysis distance, angle of progression [46,47], and midline angle in prolonged dystocic labor has not been extensively studied in the literature.

Nevertheless, this ultrasound parameter, either independently or in combination with other pelvimetric ultrasound measurements, appears to be particularly valuable in evaluating dystocic labor. Furthermore, the use of ultrasound, especially in assessing asynclitism, has been associated with reduced medical-legal issues, including fewer claims, decreased liability, and less litigation [48,49].

### 4.2. Asynclitism Degree, Geometric Dystocia and AIDA

Malgieri provided an explanation of artificial intelligence’s use during labor [50]. Malvasi and Malgieri et al. [13] used artificial intelligence and machine learning algorithms highlighting the need to measure the degree of asynclitism in prolonged dystocic labor. According to this study, a significant rise in the rate of cesarean birth was associated with an asynclitism degree greater than 70 mm, as shown in Figure 2, and AIDA classes 3 and 4. 

AIDA [13] uses machine learning algorithms to analyze these four geometric parameters collectively, creating a more nuanced understanding of the fetal head’s orientation, position and descent. The algorithm categorizes cases into five different AIDA classes based on the values of these parameters, with each class associated with different probabilities of spontaneous delivery, operative vaginal delivery, or the need for intrapartum cesarean delivery.

Specifically, for asynclitism degree, the AIDA method and the five different AIDA classes identified based on the AD values and cut-off range were associated with a higher likelihood of intrapartum cesarean delivery. This cut-off helps in stratifying risk and guiding clinical decision-making by substituting numerical values for the adjectives describing physiological, mild, or pathological asynclitism. Integrating AD into the AIDA system represents a significant advancement over traditional methods of assessing labor progress for the following reasons: (a) objectivity: it provides a quantitative measure of asynclitism, reducing reliance on subjective digital examinations; (b) comprehensive assessment: by considering AD alongside other parameters, AIDA offers a more complete picture of the geometric relationships in the birth canal; (c) predictive power: the inclusion of AD enhances the algorithm’s ability to predict labor outcomes, potentially allowing for earlier intervention when necessary; (d) standardization: it contributes to a more standardized approach to diagnosing and managing labor in the case of “geometric dystocia”; and (e) personalized care: the detailed information provided by AIDA, including AD measurements, allows for more tailored management of individual cases.

The progression distance was measured in AIDA by means of the head-to-symphysis distance (HSD) but could also be measured by means of the head-to-perineum distance (HPD) [51]. 

By incorporating asynclitism degree into its analysis, AIDA represents a promising and helpful decision support system for defining and managing geometric dystocia. This method has the potential to improve labor management, reduce unnecessary interventions, and ultimately enhance maternal and fetal outcomes.

### 4.3. Highlights from the AIDA2

The findings of this study represent a significant step forward in the application of artificial intelligence to intrapartum care, particularly in the management of labor dystocia. By integrating multiple geometric parameters measured through intrapartum ultrasonography with machine learning algorithms, the AIDA method offers a novel approach to assessing labor progress and predicting delivery outcomes. The results not only validate the importance of AD in labor progression but also demonstrate the potential of AI-assisted decision support tools in obstetric practice. 

One of the most striking findings of this study is the identification of specific cut-off ranges for AD associated with an increased likelihood of ICD. The range between 65.5 mm and 70.5 mm appears to represent a critical threshold beyond which the risk of cesarean delivery significantly increases. This quantitative criterion provides clinicians with a more objective basis for risk assessment, potentially enhancing the precision of clinical decision-making during prolonged labor. The fact that this cut-off range emerged from the analysis of AD in combination with other parameters (AoP, HSD, MLA) underscores the complex interplay of factors influencing labor progression and highlights the value of a multifaceted approach to labor assessment.

The weak to very weak correlations observed between AD and the other geometric parameters (AoP, HSD, MLA) suggest that asynclitism operates somewhat independently in affecting labor progress. This finding challenges the traditional view of labor mechanics and emphasizes the importance of measuring AD as a distinct parameter. It also highlights the potential limitations of relying solely on conventional measures like the angle of progression or head-perineum distance in assessing labor progress, particularly in cases of suspected asynclitism.

The high accuracy of the AIDA algorithm in predicting delivery outcomes, particularly for AIDA classes 0 and 4, is remarkable. The 100% agreement between algorithm predictions and physician-determined outcomes in these classes demonstrates the potential of AI to provide reliable decision support in clear-cut cases. For AIDA class 0, where all parameters were in the green zone, the consistent prediction of non-ICD outcomes suggests that the algorithm could help identify cases where intervention might be safely avoided, potentially reducing unnecessary cesarean deliveries. Conversely, the accurate prediction of ICD in AIDA class 4 cases could facilitate earlier decision-making for cesarean delivery, potentially improving maternal and fetal outcomes by reducing the duration of prolonged, unproductive labor.

The more nuanced results observed in AIDA class 3, where three parameters were in the red or yellow zone and one was in the green zone, reflect the complexity of clinical decision-making in cases with mixed indicators. The variability in algorithm predictions for this class underscores the continued importance of clinical judgment and the potential for AI to serve as a supportive rather than a decisive tool in such cases. It also highlights an area for future refinement of the algorithm, perhaps through the incorporation of additional clinical parameters or the development of more sophisticated machine learning models capable of handling these complex cases.

The finding that the type of asynclitism (anterior or posterior) is secondary to its degree and other parameters in predicting outcomes challenges some traditional views and suggests a need for a more nuanced understanding of fetal positioning in labor. This result aligns with the recent literature questioning the clinical significance of occiput posterior position as an independent risk factor for cesarean delivery and emphasizes the importance of considering multiple factors in assessing labor progress.

The AIDA method’s integration of multiple geometric parameters measured through intrapartum ultrasonography represents a significant advancement in the objective assessment of labor progress. Traditional methods of labor assessment, such as digital vaginal examinations, are known to be subjective and prone to inter-observer variability. By providing a more standardized and quantitative approach, the AIDA method has the potential to reduce this variability and improve the consistency of clinical decision-making across different practitioners and healthcare settings.

Moreover, the use of machine learning algorithms to analyze these parameters and predict outcomes represents a paradigm shift in obstetric care. The ability of these algorithms to identify complex patterns and relationships that may not be immediately apparent to human observers offers the potential for more accurate risk stratification and personalized management strategies. This approach aligns with the growing trend towards precision medicine in other areas of healthcare and could lead to more tailored interventions based on individual patient characteristics and labor patterns.

### 4.4. Potential Clinical Implications

The potential clinical implications of this study are significant. By providing a more objective and comprehensive assessment of labor progress, the AIDA method could enhance clinical decision-making in several ways:

1. Early identification of high-risk cases: accurate prediction of ICD likelihood in AIDA class 4 cases could facilitate timely intervention, potentially reducing the risks associated with prolonged labor.
2. Reduction in unnecessary interventions: reliable identification of low-risk cases (AIDA class 0) could help avoid unnecessary cesarean deliveries.
3. Personalized labor management: the integration of multiple parameters allows for a more nuanced assessment of individual cases, potentially leading to more tailored management strategies.
4. Targeted interventions: understanding the degree of asynclitism could guide the use of specific interventions aimed at promoting fetal head rotation and descent, such as maternal positioning or manual rotation techniques.
5. Optimizing the timing of interventions: in cases of severe asynclitism with other unfavorable parameters, earlier decision-making for cesarean delivery could potentially reduce the risks associated with prolonged, unproductive labor.
6. Reducing unnecessary interventions: conversely, in cases where AD and other parameters are favorable, clinicians might feel more confident in allowing labor to continue, potentially reducing unnecessary interventions.
7. Improved communication: the quantitative nature of the AIDA predictions could facilitate clearer communication between healthcare providers and patients regarding the likelihood of different delivery outcomes.
8. Enhanced training: the AIDA method could serve as an educational tool for training obstetricians and midwives in interpreting intrapartum ultrasound findings and assessing labor progress.

However, the implementation of AI-assisted decision support tools in clinical practice also raises important ethical and practical considerations. There is a need to ensure that these tools enhance rather than replace clinical judgment and that they are used in a way that respects patient autonomy and informed decision-making. The potential for algorithmic bias, particularly if the training data are not representative of diverse patient populations, must also be carefully considered and addressed.

The study’s findings also have implications for our understanding of labor mechanics and the pathophysiology of dystocia. The importance of asynclitism degree as a predictor of labor outcomes suggests that more attention should be paid to this aspect of fetal positioning in both research and clinical practice. Future studies might explore the biomechanical factors contributing to asynclitism and investigate potential interventions to correct or mitigate its effects on labor progress.

### 4.5. Study Limitations and Future Research Directions

The study’s focus on nulliparous women with singleton pregnancies receiving neuraxial analgesia during prolonged second stage labor is both a strength and a limitation. While it allows for a detailed examination of a specific high-risk group, it also limits the generalizability of the findings to other patient populations. Future research should explore the applicability of the AIDA method to multiparous women, those without analgesia, and cases of shorter labor duration to establish its broader clinical utility.

The retrospective nature of the study, while allowing for the analysis of a substantial dataset, introduces potential biases and limits causal inferences. Prospective validation studies will be crucial to confirm the predictive accuracy of the AIDA algorithm in real-time clinical settings and to assess its impact on clinical decision-making and patient outcomes. Such studies should ideally be conducted across multiple centers and diverse patient populations to ensure the robustness and generalizability of the findings.

The reliance on specific ultrasound equipment and AI algorithms raises questions about the reproducibility of these findings in settings with different technological capabilities. As intrapartum ultrasonography becomes more widespread, it will be important to establish standardized protocols for measurement and ensure that the AIDA method can be effectively implemented across various hardware and software platforms. This may require collaboration between researchers, clinicians, and technology developers to create user-friendly interfaces and ensure seamless integration with existing clinical workflows.

The study presents several limitations that merit consideration. The small sample size of 135 participants restricts statistical power and the detection of subtle effects. Its retrospective nature introduces potential biases in data collection and interpretation. The focus on nulliparous women with specific conditions limits generalizability. Data from three Italian hospitals may not represent diverse healthcare settings, potentially affecting external validity. While the study innovatively uses AI with echography to measure four geometric parameters (HSD, AD, AoP, MLA), this approach introduces new challenges. The accuracy and reproducibility of AI-assisted measurements in varied clinical settings and with different ultrasound equipment remain uncertain. There exists a potential for measurement inconsistencies due to variations in image quality or fetal positioning. The AI algorithm’s performance might be overfitted to this specific dataset, calling into question its efficacy across diverse populations. This study also lacks long-term outcome data and a prospective validation cohort, limiting conclusions about the algorithm’s real-world clinical impact. Additionally, the integration of AI-assisted echography into routine practice may face technological and training barriers, potentially limiting its widespread adoption and the generalizability of the study’s findings.

Several challenges and considerations must be addressed:

1. Standardization of measurement techniques: to ensure reproducibility and comparability of AD measurements across different settings, standardized protocols for intrapartum ultrasonography need to be established and widely adopted.
2. Training and education: widespread implementation of AD assessment would require training programs for clinicians to ensure competence in both the technical aspects of measurement and the interpretation of results.
3. Technology integration: the successful implementation of AI-assisted tools like AIDA will depend on their seamless integration into existing clinical workflows and electronic health record systems.
4. Validation in diverse populations: While this study provides valuable insights, larger-scale studies across diverse patient populations are needed to validate the predictive value of AD and refine the AIDA algorithm.
5. Ethical considerations: as with any AI-assisted decision support tool, careful consideration must be given to issues of transparency, accountability, and the potential for algorithmic bias.

Future research directions should include:

1. Large-scale, prospective validation studies across diverse healthcare settings and patient populations.
2. Investigation of the AIDA method’s performance in different clinical scenarios, including multiparous women and labors of shorter duration.
3. Integration of additional clinical parameters (e.g., maternal characteristics, labor progress metrics, and maternal and fetal complications) to potentially enhance the predictive accuracy of the algorithm.
4. Evaluation of the impact of AIDA implementation on clinical outcomes, including cesarean delivery rates, maternal and neonatal morbidity, and patient satisfaction.
5. Cost-effectiveness analyses to assess the economic implications of implementing the AIDA method in routine clinical practice.
6. Exploration of the potential role of the AIDA method in reducing healthcare disparities by providing more objective assessment tools.

## 5. Conclusions

This study highlights the significant role of asynclitism degree in labor progression and outcomes. The integration of AD measurement into the AIDA algorithm represents a novel approach to labor assessment that has the potential to enhance clinical decision-making and improve outcomes in cases of suspected dystocia. By quantifying asynclitism degree and integrating it into predictive models, we open new avenues for research and clinical practice that may ultimately lead to safer, more individualized care for women in labor. The AIDA method represents a significant advancement in the application of artificial intelligence to intrapartum care. By providing a more objective and comprehensive assessment of labor progress, with a particular focus on asynclitism degree, it has the potential to enhance clinical decision-making and improve outcomes in cases of suspected dystocia. However, its integration into clinical practice should be approached cautiously, with ongoing evaluation and refinement based on larger-scale studies and real-world implementation experiences. As we move towards an era of AI-assisted healthcare, it is crucial that we continue to critically evaluate these helpful and promising tools, ensuring that they support rather than supplant clinical expertise and that they ultimately contribute to improved patient care and outcomes.

## Figures and Tables

**Table 1:** Demographic and clinical characteristics of study participants (N = 135).

Characteristic	Mean	Standard Deviation
Age (years)	31.62	5.28
Gestational age (weeks)	40.16	1.02
Gestational age (days)	283.09	7.15
BMI	27.52	2.95
Neonatal weight (grams)	3926.68	309.66
Apgar Score (1 min)	6.65	1.22
Apgar Score (5 min)	8.74	1.12

**Table 2:** Descriptive statistics for geometric parameters (N = 135).

Characteristic	Minimum	Maximum	Mean	Standard Deviation
Head-Symphysis Distance (HSD) (mm)	10	51	21.47	9.265
Asynclitism Degree (AD) (mm)	4	95	60.18	18.866
Midline Angle (MLA) (°)	26	90	62.59	14.986
Angle of Progression (AoP) (°)	72	192	122.75	27.454

**Table 3:** Pearson’s correlations of asynclitism degree (AD) with other parameters for 135 study participants.

Pearson’s Correlations		PC		*p*
Apgar Score at 1 min.	Apgar Score at 5 min.	0.8	very strong	8.42 × 10^−32^
Angle of Progression	Asynclitism Degree	0.36	weak	0.00002
Head-Symphysis Distance	Asynclitism Degree	0.18	very weak	0.03
Midline angle	Asynclitism Degree	0.14	very weak	0.09
Apgar Score at 1 min.	Asynclitism Degree	−0.2	weak	0.02
Apgar Score at 5 min.	Asynclitism Degree	−0.19	very weak	0.02

* *p* < 0.05; ** *p* < 0.01; *** *p* < 0.001.

**Table 4:** The hierarchy of asynclitism degree for each of the six machine learning algorithms used in AIDA [13].

ALGORITHM	AD
MLP	fourth
Random Forest	third
SVM	second
XGBoost	second
Logistic Regression	fourth
Decision Tree	third

**Table 5:** Cut-off values for asynclitism degree (AD) and three other associated geometric parameters, AoP, HSD, and MLA, in predicting intrapartum cesarean delivery (ICD) and no ICD.

	AoP (°)	AD (mm)	HSD (mm)
AD–AoP	<101.5	≥67	
	≥144.5		
AD–HSD		≥70.5	≥19.5
AD–MLA		≥65.5	

**Table 6:** Confusion matrix and performances of AIDA algorithms [13], random forest (RF), support vector machine (SVM), and multi-layer perceptron (MLP), in predicting delivery outcomes for AIDA classes 0, 3, and 4. The analysis encompasses a total of 86 patients and 149 predictions.

AIDA CLASS	Algorithms	Accuracy	PPV	NPV	Recall	Specificity	F1 Score	TP	FP	FN	TN
CLASS 0	RF	1	NA	1	NA	1	NA	0	0	0	68
	SVM	0.9853	0.00	1	NA	0.9853	NA	0	1	0	67
	MLP	1	NA	1	NA	1	NA	0	0	0	68
CLASS 3	RF	0.920	0.9167	1	1	0.3333	0.9565	44	4	0	2
	SVM	0.880	0.880	NA	1	0.00	0.9362	44	6	0	0
	MLP	0.780	0.8667	0.00	0.8864	0.00	0.8764	39	6	5	0
CLASS 4	RF	1	1	NA	1	NA	1	31	0	0	0
	SVM	1	1	NA	1	NA	1	31	0	0	0
	MLP	0.9677	1	0.00	0.9677	NA	0.9836	30	0	1	0

**Table 7:** Detailed breakdown of predictions for each patient in AIDA class 3, encompassing 30 patients and 50 predictions. The data were organized by asynclitism type (anterior or posterior) and include the number of predictions for each patient. The table presents the delivery outcome, AIDA parameter classifications (AD, AoP, MLA, SPD), and the predictions made by each of the three algorithms (SVM, RF, MLP). To facilitate interpretation, the algorithm predictions are color-coded: green indicates agreement with the actual delivery outcome, while red signifies disagreement. This comprehensive presentation allows for a nuanced analysis of the algorithms’ performance in relation to asynclitism type and individual patient characteristics within AIDA class 3.

Number of Predictions	ID Patient	Delivery Outcome	AIDA AD	AIDA AoP	AIDA MLA	AIDA SPD	Predicted Outcome (SVM)	Predicted Outcome (RF)	Predicted Outcome (MLP)
**Anterior Asynclitism**									
2	26	ICD	GREEN	RED	RED	RED	**ICD**	**ICD**	**ICD**
2	29	ICD	GREEN	RED	RED	RED	**ICD**	**ICD**	**ICD**
2	110	ICD	RED	RED	RED	GREEN	**ICD**	**ICD**	**ICD**
2	115	NOICD	RED	RED	RED	GREEN	**ICD**	**NOICD**	**ICD**
1	127	ICD	YELLOW	RED	GREEN	RED	**ICD**	**ICD**	**ICD**
2	127	ICD	YELLOW	RED	GREEN	RED	**ICD**	**ICD**	**NOICD**
**Posterior Asynclitism**									
1	12	ICD	GREEN	RED	RED	RED	**ICD**	**ICD**	**ICD**
1	28	ICD	GREEN	RED	RED	RED	**ICD**	**ICD**	**ICD**
1	33	ICD	GREEN	RED	RED	RED	**ICD**	**ICD**	**ICD**
3	39	ICD	GREEN	RED	RED	RED	**ICD**	**ICD**	**ICD**
1	42	ICD	GREEN	RED	RED	RED	**ICD**	**ICD**	**ICD**
2	44	ICD	GREEN	RED	YELLOW	RED	**ICD**	**ICD**	**ICD**
1	50	ICD	GREEN	RED	RED	RED	**ICD**	**ICD**	**ICD**
2	51	ICD	GREEN	RED	RED	RED	**ICD**	**ICD**	**ICD**
2	63	ICD	GREEN	RED	RED	RED	**ICD**	**ICD**	**ICD**
2	77	ICD	GREEN	RED	RED	RED	**ICD**	**ICD**	**ICD**
1	96	ICD	GREEN	RED	RED	RED	**ICD**	**ICD**	**ICD**
3	7	ICD	GREEN	RED	RED	RED	**ICD**	**ICD**	**ICD**
2	20	ICD	GREEN	RED	RED	RED	**ICD**	**ICD**	**ICD**
2	24	NOICD	GREEN	RED	RED	RED	**ICD**	**ICD**	**ICD**
2	43	ICD	GREEN	RED	RED	RED	**ICD**	**ICD**	**ICD**
1	46	ICD	GREEN	RED	RED	RED	**ICD**	**ICD**	**ICD**
2	118	ICD	GREEN	RED	RED	RED	**ICD**	**ICD**	**ICD**
1	59	ICD	RED	RED	GREEN	RED	**ICD**	**ICD**	**ICD**
1	88	ICD	RED	RED	GREEN	RED	**ICD**	**ICD**	**ICD**
1	99	ICD	RED	GREEN	RED	RED	**ICD**	**ICD**	**ICD**
2	100	NOICD	RED	RED	RED	GREEN	**ICD**	**ICD**	**ICD**
1	126	ICD	RED	RED	RED	GREEN	**ICD**	**ICD**	**ICD**
1	135	ICD	RED	RED	RED	GREEN	**ICD**	**ICD**	**NOICD**
1	124	ICD	YELLOW	RED	GREEN	RED	**ICD**	**ICD**	**ICD**
2	38	ICD	YELLOW	RED	GREEN	RED	**ICD**	**ICD**	**NOICD**

**Table 8:** Detailed analysis of predictions for patients in AIDA class 0, comprising 38 patients with a total of 68 predictions. The data were organized by asynclitism type (anterior or posterior) and include the frequency of predictions for each patient. The table displays the actual delivery outcome, AIDA parameter classifications (AD, AoP, MLA, SPD), and the predictions made by each of the three algorithms (SVM, RF, MLP). For easy interpretation, the algorithm predictions are color-coded: green indicates agreement with the actual delivery outcome, while red denotes disagreement. This comprehensive presentation enables a thorough examination of the algorithms’ performance in relation to asynclitism type and individual patient characteristics within AIDA class 0, where all four parameters are in the green zone.

Number of Predictions	ID Patient	Delivery Outcome	AIDA AD	AIDA AoP	AIDA MLA	AIDA SPD	Predicted Outcome (SVM)	Predicted Outcome (RF)	Predicted Outcome (MLP)
**Anterior Asynclitism**									
3	3	NOICD	GREEN	GREEN	GREEN	GREEN	**NOICD**	**NOICD**	**NOICD**
1	17	NOICD	GREEN	GREEN	GREEN	GREEN	**NOICD**	**NOICD**	**NOICD**
2	21	NOICD	GREEN	GREEN	GREEN	GREEN	**NOICD**	**NOICD**	**NOICD**
2	23	NOICD	GREEN	GREEN	GREEN	GREEN	**NOICD**	**NOICD**	**NOICD**
2	30	NOICD	GREEN	GREEN	GREEN	GREEN	**NOICD**	**NOICD**	**NOICD**
2	35	NOICD	GREEN	GREEN	GREEN	GREEN	**NOICD**	**NOICD**	**NOICD**
3	37	NOICD	GREEN	GREEN	GREEN	GREEN	**NOICD**	**NOICD**	**NOICD**
1	40	NOICD	GREEN	GREEN	GREEN	GREEN	**ICD**	**NOICD**	**NOICD**
1	45	NOICD	GREEN	GREEN	GREEN	GREEN	**NOICD**	**NOICD**	**NOICD**
3	48	NOICD	GREEN	GREEN	GREEN	GREEN	**NOICD**	**NOICD**	**NOICD**
3	87	NOICD	GREEN	GREEN	GREEN	GREEN	**NOICD**	**NOICD**	**NOICD**
1	105	NOICD	GREEN	GREEN	GREEN	GREEN	**NOICD**	**NOICD**	**NOICD**
1	109	NOICD	GREEN	GREEN	GREEN	GREEN	**NOICD**	**NOICD**	**NOICD**
1	125	NOICD	GREEN	GREEN	GREEN	GREEN	**NOICD**	**NOICD**	**NOICD**
1	6	NOICD	GREEN	GREEN	GREEN	GREEN	**NOICD**	**NOICD**	**NOICD**
1	14	NOICD	GREEN	GREEN	GREEN	GREEN	**NOICD**	**NOICD**	**NOICD**
2	19	NOICD	GREEN	GREEN	GREEN	GREEN	**NOICD**	**NOICD**	**NOICD**
2	41	NOICD	GREEN	GREEN	GREEN	GREEN	**NOICD**	**NOICD**	**NOICD**
3	62	NOICD	GREEN	GREEN	GREEN	GREEN	**NOICD**	**NOICD**	**NOICD**
2	75	NOICD	GREEN	GREEN	GREEN	GREEN	**NOICD**	**NOICD**	**NOICD**
1	95	NOICD	GREEN	GREEN	GREEN	GREEN	**NOICD**	**NOICD**	**NOICD**
1	103	NOICD	GREEN	GREEN	GREEN	GREEN	**NOICD**	**NOICD**	**NOICD**
1	134	NOICD	GREEN	GREEN	GREEN	GREEN	**NOICD**	**NOICD**	**NOICD**
**Posterior Asynclitism**									
1	1	NOICD	GREEN	GREEN	GREEN	GREEN	**NOICD**	**NOICD**	**NOICD**
3	5	NOICD	GREEN	GREEN	GREEN	GREEN	**NOICD**	**NOICD**	**NOICD**
2	27	NOICD	GREEN	GREEN	GREEN	GREEN	**NOICD**	**NOICD**	**NOICD**
3	49	NOICD	GREEN	GREEN	GREEN	GREEN	**NOICD**	**NOICD**	**NOICD**
1	56	NOICD	GREEN	GREEN	GREEN	GREEN	**NOICD**	**NOICD**	**NOICD**
3	80	NOICD	GREEN	GREEN	GREEN	GREEN	**NOICD**	**NOICD**	**NOICD**
1	108	NOICD	GREEN	GREEN	GREEN	GREEN	**NOICD**	**NOICD**	**NOICD**
3	130	NOICD	GREEN	GREEN	GREEN	GREEN	**NOICD**	**NOICD**	**NOICD**
1	16	NOICD	GREEN	GREEN	GREEN	GREEN	**NOICD**	**NOICD**	**NOICD**
2	31	NOICD	GREEN	GREEN	GREEN	GREEN	**NOICD**	**NOICD**	**NOICD**
2	54	NOICD	GREEN	GREEN	GREEN	GREEN	**NOICD**	**NOICD**	**NOICD**
2	81	NOICD	GREEN	GREEN	GREEN	GREEN	**NOICD**	**NOICD**	**NOICD**
2	89	NOICD	GREEN	GREEN	GREEN	GREEN	**NOICD**	**NOICD**	**NOICD**
1	90	NOICD	GREEN	GREEN	GREEN	GREEN	**NOICD**	**NOICD**	**NOICD**
1	132	NOICD	GREEN	GREEN	GREEN	GREEN	**NOICD**	**NOICD**	**NOICD**

**Table 9:** Comprehensive analysis of predictions for patients in AIDA class 4, encompassing 18 patients with a total of 31 predictions. The data were organized by asynclitism type (anterior or posterior) and include the frequency of predictions for each patient. The table displays the actual delivery outcome, AIDA parameter classifications (AD, AoP, MLA, SPD), and the predictions made by each of the three algorithms (SVM, RF, MLP). For clear interpretation, the algorithm predictions are color-coded: green indicates agreement with the actual delivery outcome, while red signifies disagreement. This detailed presentation allows for an in-depth examination of the algorithms’ performance in relation to asynclitism type and individual patient characteristics within AIDA class 4, where all four parameters are in the red or yellow zones.

Number of Predictions	ID Patient	Delivery Outcome	AIDA AD	AIDA AoP	AIDA MLA	AIDA SPD	Predicted Outcome (SVM)	Predicted Outcome (RF)	Predicted Outcome (MLP)
**Anterior Asynclitism**									
1	74	ICD	RED	RED	RED	RED	**ICD**	**ICD**	**ICD**
2	83	ICD	RED	RED	RED	RED	**ICD**	**ICD**	**ICD**
1	2	ICD	YELLOW	RED	YELLOW	RED	**ICD**	**ICD**	**ICD**
3	4	ICD	YELLOW	RED	RED	RED	**ICD**	**ICD**	**ICD**
4	22	ICD	RED	RED	RED	RED	**ICD**	**ICD**	**ICD**
1	67	ICD	RED	RED	RED	RED	**ICD**	**ICD**	**ICD**
1	121	ICD	RED	RED	RED	RED	**ICD**	**ICD**	**ICD**
**Posterior Asynclitism**									
1	102	ICD	RED	RED	RED	RED	**ICD**	**ICD**	**ICD**
3	60	ICD	YELLOW	RED	RED	RED	**ICD**	**ICD**	**ICD**
2	69	ICD	YELLOW	RED	RED	RED	**ICD**	**ICD**	**ICD**
1	84	ICD	RED	RED	RED	RED	**ICD**	**ICD**	**ICD**
1	85	ICD	RED	RED	RED	RED	**ICD**	**ICD**	**ICD**
1	93	ICD	RED	RED	RED	RED	**ICD**	**ICD**	**ICD**
3	117	ICD	RED	RED	RED	RED	**ICD**	**ICD**	**ICD**
3	18	ICD	RED	RED	RED	RED	**ICD**	**ICD**	**ICD**
1	71	ICD	RED	RED	RED	RED	**ICD**	**ICD**	**ICD**
1	78	ICD	RED	RED	RED	RED	**ICD**	**ICD**	**NOICD**
1	114	ICD	RED	RED	RED	RED	**ICD**	**ICD**	**ICD**

## Data Availability

The authors of the study are the custodians of the data in anonymous form, which could possibly be provided to anyone who makes a motivated and reasoned request.
